# Supplementary material for: Acetophenone-Based 3,4-Dihydropyrimidine-2(1H)-Thione as Potential Inhibitor of Tyrosinase and Ribonucleotide Reductase: Facile Synthesis, Crystal Structure, In-Vitro and In-Silico Investigations
Source: Int J Mol Sci. 2022 Oct 29;23(21):13164. doi: 10.3390/ijms232113164 (PMC9658835; doi:10.3390/ijms232113164)
Supplement: Supplementary file 1 [file ijms-23-13164-s001.zip › ijms-1996294-supplementary.pdf]

Supplementary File

Article

Acetophenone-Based 3,4-Dihydropyrimidine-2(1H)-Thione as Potential Inhibitor of Tyrosinase and Ribonucleotide Reductase: Facile Synthesis, Crystal Structure, In-Vitro and In-Silico Investigations

Aamer Saeed [1,*], Syeda Abida Ejaz [2,*], Aqsa Khalid [1], Pervaiz Ali Channar [3], Mubashir Aziz [2], Qamar Abbas [4], Tanveer A. Wani [5], Nawaf A. Alsaif [5], Mohammed M. Alanazi [5], Abdullah M. Al-Hossaini [5], Nojood Altwaijry [6], Seema Zargar [6], Muawya Elhadi [7] and Tuncer Hökelek [8]

[1] Department of Chemistry, Quaid-i-Azam University, Islamabad 45320, Pakistan
[2] Department of Pharmaceutical Chemistry, Faculty of Pharmacy, The Islamia University of Bahawalpur, Bahawalpur 63100, Pakistan
[3] Department of Basic Sciences and Humanities, Faculty of of Information Science and Humanities, Dawood University of Engineering and Technology, Karachi 74800, Pakistan
[4] Department of Biology, College of Science, University of Bahrain, Sakhir Campus, Zallaq 32038, Bahrain
[5] Department of Pharmaceutical Chemistry, College of Pharmacy, King Saud University, P.O. Box 2457, Riyadh 11451, Saudi Arabia
[6] Department of Biochemistry, College of Science, King Saud University, P.O. Box 22452, Riyadh 11451, Saudi Arabia
[7] Department of Physics, Faculty of Science and Humanities, Ed Dawadmi, Shaqra University, Shaqra 11961, Saudi Arabia
[8] Department of Physics, Faculty of Engineering, Hacettepe University, Beytepe, Ankara 06800, Turkey
* Correspondence: aamersaeed@yahoo.com (A.S.); abida.ejaz@iub.edu.pk (S.A.E.);
Tel.: +92-51-9064-2128 (A.S.); +92-062-9250245 (S.A.E.); Fax: +92-51-9064-2241 (A.S.); +92-062-9250245 (S.A.E.)

Mushroom tyrosinase inhibition assay

In detail, 140 µL of phosphate buffer (20 mM, pH 6.8), 20 µL of mushroom tyrosinase (30 U/mL) and 20 µL of the inhibitor solution were placed in the wells of a 96-well micro plate. After pre-incubation for 10 min at room temperature, 20 µL of L-DOPA (3,4-dihydroxyphenylalanine, Sigma Chemical, USA) (0.85 mM) was added and the assay plate was further incubated at 25 °C for 20 min. Afterward the absorbance of dopachrome was measured at 475 nm using a micro plate reader (OPTI Max, Tunable). Kojic acid was used as a reference inhibitor and phosphate buffer was used as a negative control. The amount of inhibition by the test compounds was expressed as the percentage of concentration necessary to achieve 50 % inhibition (IC_{50}). Each concentration was analyzed in three independent experiments. IC_{50} values were calculated by nonlinear regression using GraphPad Prism 5.0 [1].

The % of Inhibition of tyrosinase was calculated as following

$$\text{Inhibition (\%)} = [(B-S)/B] \times 100$$

Here, the B and S are the absorbance's for the blank and samples.

Hirshfeld surface – HS Studies

The white surface in the HS results represents contacts with distances equal to the sum of van der Waals radii(Figure 4), in the same way the red colour indicate distances shorter (in close touch) and blue colour represents longer distances (distinct contact) than the van der Waals radii, respectively [2]. The bright-red dots explains their role as donor and/or acceptor; they also appear as blue and red regions on the HS mapped over electrostatic potential [3], as illustrated in Figure 4. (C). Positive electrostatic potential i.e. hydrogen-bond donors are indicated by blue, whereas negative electrostatic potential is

indicated by red representing hydrogen-bond acceptors. The HS shape-index is a tool for visualising π ... π stacking by the presence of adjacent red and blue triangles; the absence of nearby red and/or blue triangles indicates no π ... π interactions exist. The figure given in the main manuscript showed that there is no π ... π interactions in the data (I).

Figure 5a depicts the overall two-dimensional fingerprint plot, while Figure 5b—j depicts the fingerprint plots defined into H ••• H, H ••• S/S ••• H, H ••• C/C ••• H, H ••• O/O••• H, S ••• C/C ••• S, O ••• C/C... O, H ••• N/N... H, S ••• N/N. The most important interaction is H ••• H, which accounts for 59.5 percent of the overall crystal packing and is represented in Figure 5b as widely scattered points of high density due to the considerable hydrogen content of the molecule with the tip at de = di = 1.17Å. The fingerprint plots demarcated into H ••• S/S ••• H, Figure 5c, contacts with 16.1 percent contribution to the HS emerge from the H ••• S/S ••• H contacts (Table 3) and are regarded as a pair of spikes with the points at de + di = 2.40Å. The pairs of distinctive wings in the fingerprint plot delimited into H ••• C/C ••• H connections (13.1 percent contribution to the HS) have sharp and spread distributions of points with the tips at de + di = 3.0Å3 and de + di = 3.01Å, respectively, in the absence of C—H ••• interactions. The symmetrical distribution of points (9.2% contribution, Figure 5e) in the fingerprint plot defined into H ••• O/O ••• H contacts (Table 3) with the tips at de + di = 2.23Å. Finally, the S ••• C/C ••• S (0.6 percent, Fig. 4f), O ••• C/C ••• O (0.6 percent, Figure 5g), H ••• N/N ••• H (0.6 percent, Figure 5h), S ••• N/N ••• S (0.2 percent, Figure S2i), and C... C (0.1 percent, Figure 5j) contacts with very tiny contributions to the HS contain dispersed points.

Figures 6a—d showed Hirshfeld surface representations of the H ••• H, H ••• S/S ••• H, H ••• C/C ••• H, and H ••• O/O••• H interactions, with the function d_{norm} mapped onto the surface interactions. The closest internal and external distances (in) from certain sites on the Hirshfeld surface contacts are di and de.

The Hirsfield analysis testifies that van der Waals and hydrogen bonding was playing significant role in determining the close packing of the crystal. Moreover, H contacts were also playing important role in close packing.

Molecular Dynamic simulations

Molecular Dynamic (MD) simulations provide a dynamic understanding of the molecular interactions of protein-ligand complexes. Simulations of MD provide important insight into the binding interactions between ligand and protein. In the present investigation, MD simulations were run on a GPU-accelerated workstation utilizing Nano scale molecular dynamic (NAMD) software, and the visualization of simulated complexes was carried out using VMD 1.9.3. Both complexes were initially constructed using the CHARM-GUI solution builder, followed by solvation using the TIP3P water model. To neutralize the system, 0.15 M of the counter ion (NaCl) was introduced. The remainder MD simulation protocol was conducted using our previously reported method. The production run was executed under periodic boundary conditions (PBC) conditions for 50 ns [4].

HNMR Spectra for compound 4

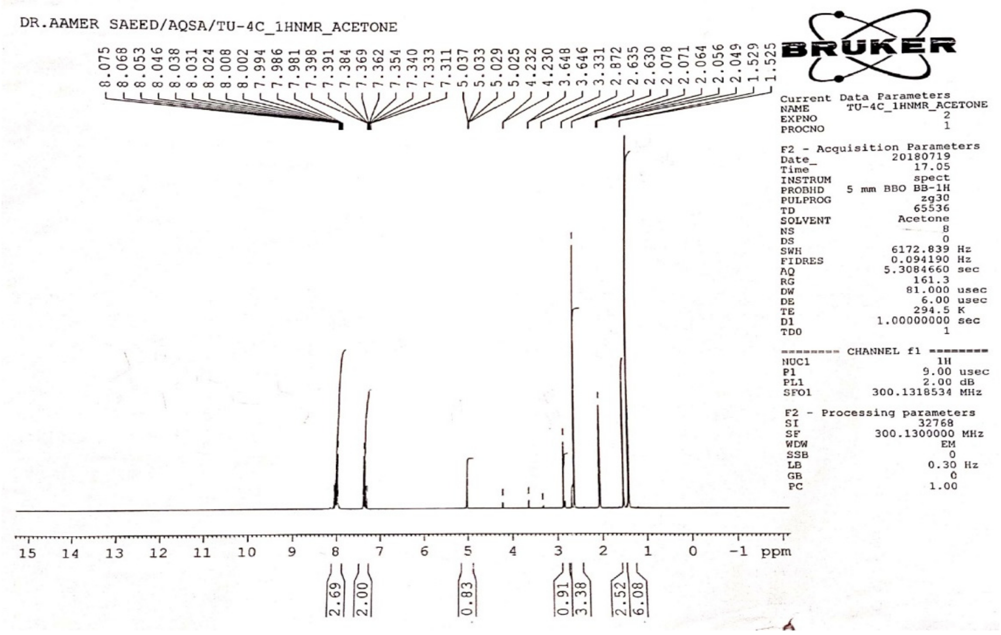

Figure S1. HNMR spectra.

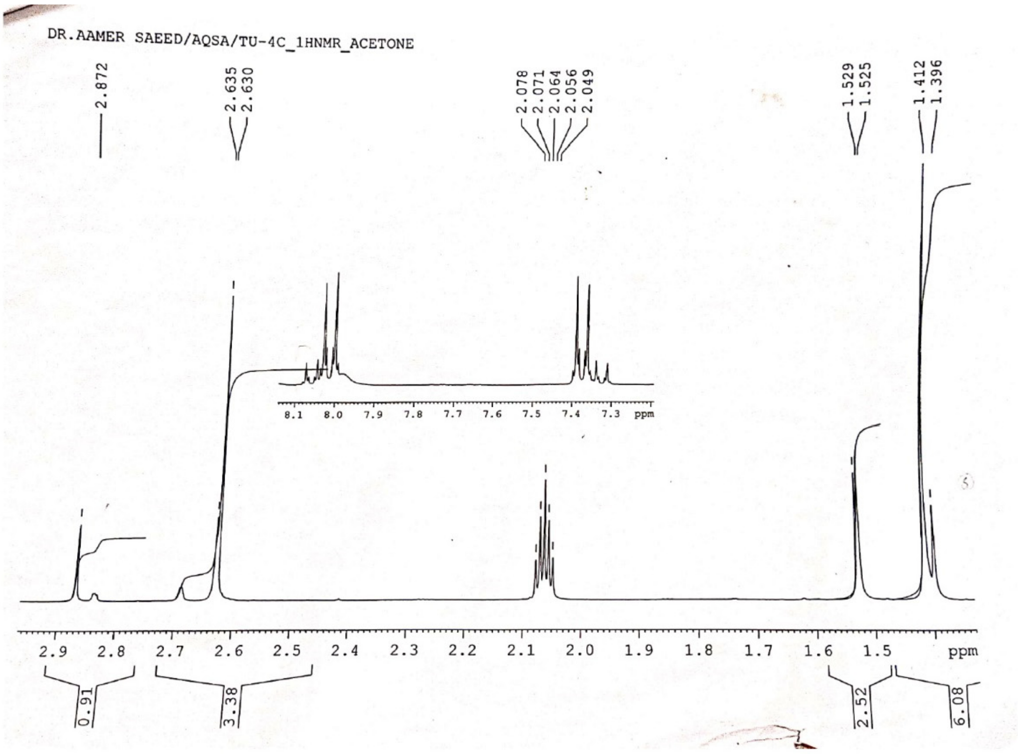

Scanned with CamScanner

Figure S2. HNMR spectra.

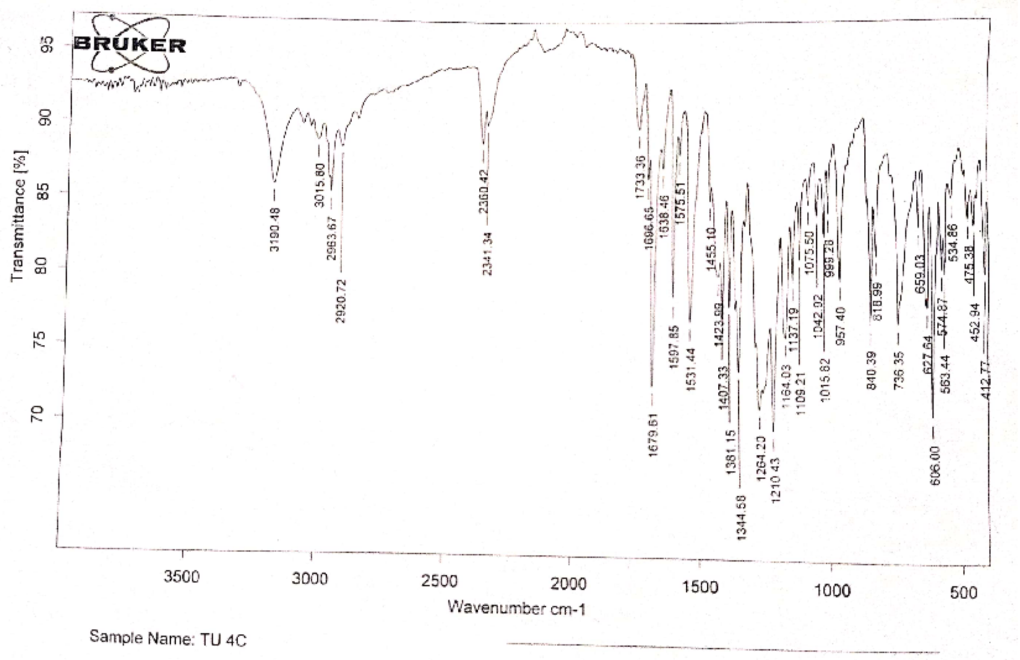

Figure S3. FTIR spectra.

[13] C NMR Spectra for compound 4

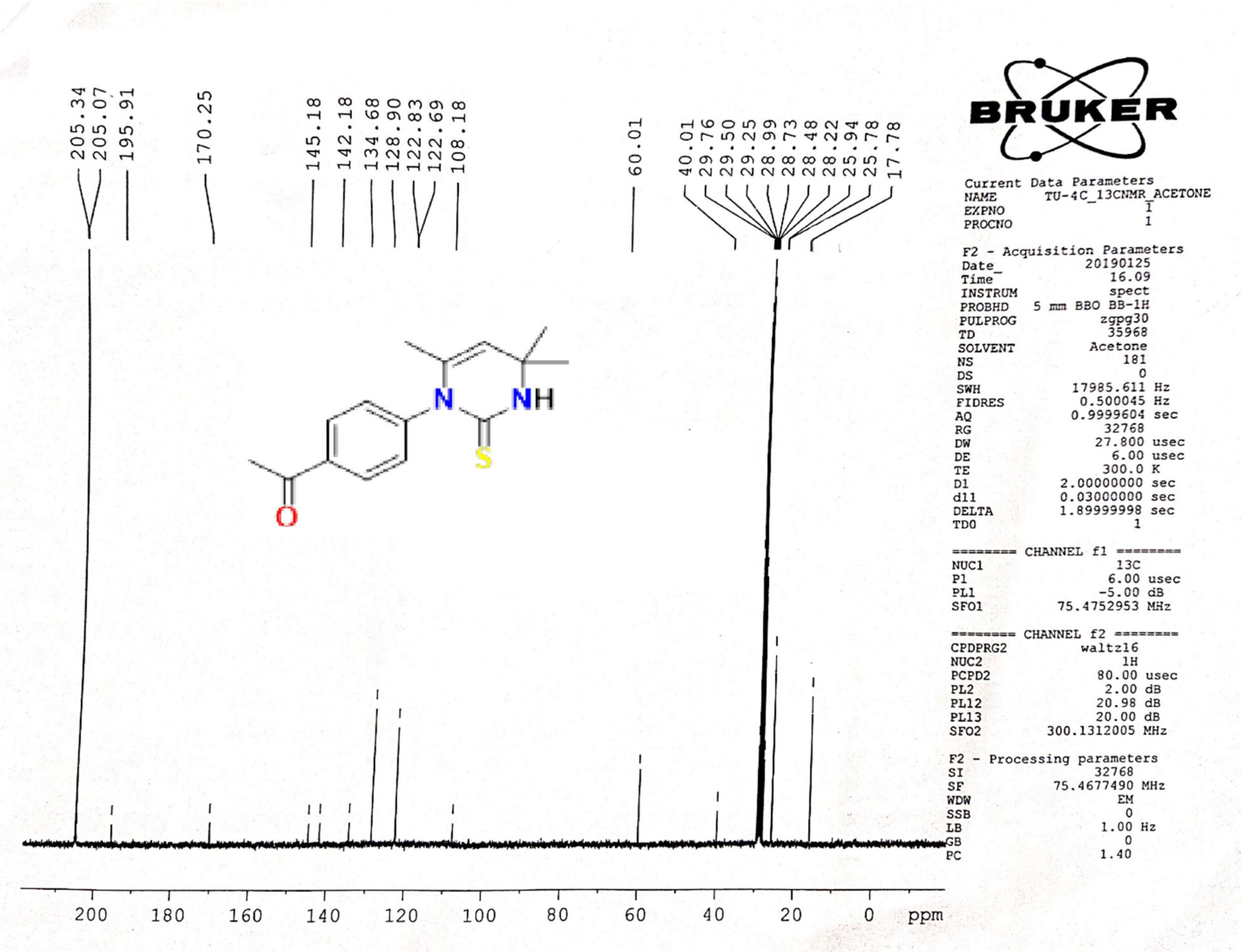

Figure S4. 13C NMR spectra for compound 4.

CIF report attached below

checkCIF/PLATON report

Structure factors have been supplied for datablock(s) shelx

THIS REPORT IS FOR GUIDANCE ONLY. IF USED AS PART OF A REVIEW PROCEDURE FOR PUBLICATION, IT SHOULD NOT REPLACE THE EXPERTISE OF AN EXPERIENCED CRYSTALLOGRAPHIC REFEREE.

No syntax errors found.   CIF dictionary   Interpreting this report

Datablock: shelx

```
Bond precision:   C-C = 0.0035 A          Wavelength=1.54184

Cell:           a=6.8262(4)        b=10.7036(9)      c=11.1396(7)
                alpha=82.423(6)    beta=75.583(5)    gamma=74.682(6)
Temperature:    173 K

                Calculated                   Reported
Volume          758.37(9)                    758.37(10)
Space group     P -1                         P -1
Hall group      -P 1                         -P 1
Moiety formula  C15 H18 N2 O S               ?
Sum formula     C15 H18 N2 O S               C15 H18 N2 O S
Mr              274.37                       274.37
Dx,g cm-3       1.202                        1.202
Z               2                            2
Mu (mm-1)       1.841                        1.841
F000            292.0                        292.0
F000'           293.39
h,k,lmax        8,13,13                      8,13,13
Nref            2959                         2883
Tmin,Tmax       0.541,0.667                  0.830,1.000
Tmin'           0.491

Correction method= # Reported T Limits: Tmin=0.830 Tmax=1.000
AbsCorr = MULTI-SCAN

Data completeness= 0.974          Theta(max)= 71.348

R(reflections)= 0.0542( 2666)                     wR2(reflections)=
                                                  0.1481( 2883)
S = 1.058                 Npar= 181
```

Figure S5. CIF/Platon report.

The following ALERTS were generated. Each ALERT has the format
test-name_ALERT_alert-type_alert-level.
Click on the hyperlinks for more details of the test.

Alert level B

```
PLAT031_ALERT_4_B Refined Extinction Parameter Within Range of ...      1.462 Sigma
```

Alert level C

```
PLAT094_ALERT_2_C Ratio of Maximum / Minimum Residual Density ....       2.95 Report
PLAT220_ALERT_2_C NonSolvent   Resd 1   C   Ueq(max)/Ueq(min) Range       3.3 Ratio
PLAT222_ALERT_3_C NonSolvent Resd 1  H   Uiso(max)/Uiso(min) Range        4.2 Ratio
PLAT230_ALERT_2_C Hirshfeld Test Diff for     C4        --C15      .      5.3 s.u.
PLAT242_ALERT_2_C Low    'MainMol' Ueq as Compared to Neighbors of         C2 Check
PLAT601_ALERT_2_C Unit Cell Contains Solvent Accessible VOIDS of .         54 Ang**3
PLAT911_ALERT_3_C Missing FCF Refl Between Thmin & STh/L=    0.600          9 Report
```

Alert level G

```
PLAT883_ALERT_1_G No Info/Value for _atom_sites_solution_primary .     Please Do !
PLAT912_ALERT_4_G Missing # of FCF Reflections Above STh/L=  0.600         66 Note
PLAT913_ALERT_3_G Missing # of Very Strong Reflections in FCF ....          2 Note
PLAT933_ALERT_2_G Number of HKL-OMIT Records in Embedded .res File          2 Note
PLAT941_ALERT_3_G Average HKL Measurement Multiplicity ..........         1.6 Low
PLAT978_ALERT_2_G Number C-C Bonds with Positive Residual Density.          8 Info
```

0 **ALERT level A** = Most likely a serious problem - resolve or explain
1 **ALERT level B** = A potentially serious problem, consider carefully
7 **ALERT level C** = Check. Ensure it is not caused by an omission or oversight
6 **ALERT level G** = General information/check it is not something unexpected

1 ALERT type 1 CIF construction/syntax error, inconsistent or missing data
7 ALERT type 2 Indicator that the structure model may be wrong or deficient
4 ALERT type 3 Indicator that the structure quality may be low
2 ALERT type 4 Improvement, methodology, query or suggestion
0 ALERT type 5 Informative message, check

Figure S6. CIF/Platon report (continued).

It is advisable to attempt to resolve as many as possible of the alerts in all categories. Often the minor alerts point to easily fixed oversights, errors and omissions in your CIF or refinement strategy, so attention to these fine details can be worthwhile. In order to resolve some of the more serious problems it may be necessary to carry out additional measurements or structure refinements. However, the purpose of your study may justify the reported deviations and the more serious of these should normally be commented upon in the discussion or experimental section of a paper or in the "special_details" fields of the CIF. checkCIF was carefully designed to identify outliers and unusual parameters, but every test has its limitations and alerts that are not important in a particular case may appear. Conversely, the absence of alerts does not guarantee there are no aspects of the results needing attention. It is up to the individual to critically assess their own results and, if necessary, seek expert advice.

Publication of your CIF in IUCr journals

A basic structural check has been run on your CIF. These basic checks will be run on all CIFs submitted for publication in IUCr journals (*Acta Crystallographica*, *Journal of Applied Crystallography*, *Journal of Synchrotron Radiation*); however, if you intend to submit to *Acta Crystallographica Section C* or *E* or *IUCrData*, you should make sure that full publication checks are run on the final version of your CIF prior to submission.

Publication of your CIF in other journals

Please refer to the *Notes for Authors* of the relevant journal for any special instructions relating to CIF submission.

PLATON version of 18/05/2022; check.def file version of 17/05/2022

Figure S7. CIF/Platon report (continued).

References

[1] Ashraf, Z.; Rafiq, M.; Seo, S.-Y.; Babar, M. M., Synthesis, kinetic mechanism and docking studies of vanillin derivatives as inhibitors of mushroom tyrosinase. *Bioorganic & medicinal chemistry* **2015,** 23, (17), 5870-5880.

[2] Spackman, M. A.; McKinnon, J. J.; Jayatilaka, D., Electrostatic potentials mapped on Hirshfeld surfaces provide direct insight into intermolecular interactions in crystals. *CrystEngComm* **2008,** 10, (4), 377-388.

[3] Yüksel, B.Ş. Spectroscopic characterization (IR and NMR), structural investigation, DFT study, and Hirshfeld surface analysis of two zinc (II) 2-acetylthiophenyl-thiosemicarbazone complexes. *Journal of Molecular Structure*. **2021** 5(1229),129617.

[4] Rasheed, S.; Aziz, M.; Saeed, A.; Ejaz, S. A.; Channar, P. A.; Zargar, S.; Abbas, Q.; Alanazi, H.; Hussain, M.; Alharbi, M., Analysis of 1-Aroyl-3-[3-chloro-2-methylphenyl] Thiourea Hybrids as Potent Urease Inhibitors: Synthesis, Biochemical Evaluation and Computational Approach. International Journal of Molecular Sciences 2022, 23, (19), 11646.
